# Physiological functions of calcium signaling via Orai1 in cancer

**DOI:** 10.1186/s12576-023-00878-0

**Published:** 2023-09-27

**Authors:** Masanari Umemura, Rina Nakakaji, Yoshihiro Ishikawa

**Affiliations:** Cardiovascular Research Institute, Yokohama City University Graduate School of Medicine, Yokohama, Kanagawa Japan

**Keywords:** Calcium, Orai1, EP4, Store-operated-calcium entry (SOCE), Cancer, Cardiac fibroblast

## Abstract

Intracellular calcium (Ca^2+^) signaling regulates many cellular functions, including cell proliferation and migration, in both normal cells and cancer cells. Store-operated Ca^2+^ entry (SOCE) is a major mechanism by which Ca^2+^ is imported from the extracellular space to the intracellular space, especially in nonexcitable cells. Store-operated Ca^2+^ entry (SOCE) is also a receptor-regulated Ca^2+^ entry pathway that maintains Ca^2+^ homeostasis by sensing reduced Ca^2+^ levels in the endoplasmic reticulum (ER). In general, the activation of G protein-coupled receptors (GPCRs) or immunoreceptors, such as T-cell, B-cell and Fc receptors, results in the production of inositol 1,4,5-trisphosphate (IP_3_). IP_3_ binds to IP_3_ receptors located in the ER membrane. The, IP_3_ receptors in the ER membrane trigger a rapid and transient release of Ca^2+^ from the ER store. The resulting depletion of ER Ca^2+^ concentrations is sensed by the EF-hand motif of stromal interaction molecule (STIM), i.e., calcium sensor, which then translocates to the plasma membrane (PM). STIM interacts with Orai Ca^2+^ channel subunits (also known as CRACM1) on the PM, leading to Ca^2+^ influx from the extracellular space to increase intracellular Ca^2+^ concentrations. The physiological functions of Orai and STIM have been studied mainly with respect to their roles in the immune system. Based on numerous previous studies, Orai channels (Orai1, Orai2 and Orai3 channels) control Ca^2+^ release-activated Ca^2+^ (CRAC) currents and contribute to SOCE currents in other types of cells, including various cancer cells. There are many reports that Orai1 is involved in cell proliferation, migration, metastasis, apoptosis and epithelial–mesenchymal transition (EMT) in various cancers. We previously found that Orai1 plays important roles in cell apoptosis and migration in melanoma. Recently, we reported novel evidence of Orai1 in human oral squamous cell carcinoma (OSCC) cells and human cardiac fibroblasts (HCFs). In this review, we present multiple physiological functions of Orai1 in various cancer cells and cardiac fibroblasts, including our findings.

## Introduction

Calcium (Ca^2+^) plays important roles as a second messenger in almost all cell types. Ca^2+^ release-activated Ca^2+^ (CRAC) channels mediate a specific form of Ca^2+^ influx called store-operated Ca^2+^ entry (SOCE) that contributes to the function of many cell types [1]. SOCE was first described in salivary gland cells as a capacitive mechanism of Ca^2+^ entry [2]. Recently, SOCE has been observed in a variety of cell types, including cardiomyocytes, cardiac fibroblasts, lymphocytes, vascular endothelial and smooth muscle cells, skeletal muscle cells, melanoma cells, oral cancer cells, breast cancer cells, glioblastoma cells, cervical cancer cells and pancreatic cancer cells. SOCE is a receptor-regulated Ca^2+^ entry pathway that is activated by the depletion of intracellular Ca^2+^ stores in the endoplasmic reticulum (ER) [3].

In 2006, Feske et al. first reported that cells from patients with one form of hereditary severe combined immune deficiency (SCID) syndrome exhibit defective SOCE and CRAC channel function [4]. The authors identified the genetic defect in these patients using a combination of two unbiased genome-wide approaches. They found a new protein that they called Orai1 (also called CRACM1; the Orai are the keepers of the gates of heaven in Greek mythology), which contains four putative transmembrane segments. Orai1 constitutes the pore subunit of SOCE-activated calcium channels. SCID patients are homozygous for a single missense mutation in Orai1. Indeed, the expression of wild-type Orai1 in T cells from patients with SCID syndrome restores SOCE and CRAC currents. Therefore, the authors concluded that Orai1 is a major Ca^2+^ channel protein that is involved in SOCE, and that it facilitates an influx of Ca^2+^ from the extracellular space to the cytoplasm through the plasma membrane (PM) in various types of cells.

Orai1 is a 33 kDa protein (composed of 301 amino acids) containing four transmembrane domains, with both its N-terminus and C-terminus residing in the cytosol [4]. CRAC channels are composed of Orai1 proteins located in the PM, which form the ion-conducting pore. Orai1 has two mammalian homologs, Orai2 and Orai3, that form Ca^2+^-selective ion channels to mediate Ca^2+^ influx [5]. Furthermore, Jardin et al. described that Orai1 possesses two variants, Orai1α and Orai1β; the latter lacks 63 amino acids in the N-terminus compared to the full-length Orai1α form, and this differences confers distinct features to each variant [6]. In this review, we focus on the function of Orai1 because among the three types of Orai homologs, most reports have focused on Orai1.

The mechanism underlying SOCE is as follows. The activation of G protein-coupled receptor (Gαq) or immunoreceptors, such as T-cell, B-cell and Fc receptors, results in the production of inositol 1, 4, 5-trisphosphate (IP_3_). IP_3_ binds to IP_3_ receptors located in the membrane of the ER. The IP_3_ receptors in the ER trigger a rapid and transient release of Ca^2+^ from the ER store. The resulting depletion of ER Ca^2+^ concentrations is sensed by EF-hand motif of stromal interaction molecule (STIM)1 and STIM2, i.e., calcium sensors, which aggregate and move to the PM. In general, The EF-hand motif of STIM binds to Ca^2+^. After its translocation to the PM where Orai1 is located, STIM interacts with Orai1 Ca^2+^ channel subunits, leading to Ca^2+^ influx from extracellular space to increase intracellular Ca^2+^ concentrations.

To date, many reports have explored the role of Orai1 in cancer. There are many reports that Orai1 is related to cell migration/metastasis and proliferation regardless of cancer type. In particular, there are many reports of breast cancer, and many reports of melanoma and oral cancer are also observed. As a general trend, there are many reports that mRNA transcription and protein expression of Orai1 are more strongly expressed in cancer cells than in normal cells regardless of cancer type. There are also reports that the degree of Orai1 expression is related to clinical outcomes, as in colorectal cancer and multiple myeloma. In addition, many reports indicate that Orai1 plays an important role in SOCE regardless of cancer type, and it is consistent that some inhibitors and knockdown of Orai1 by siRNA or shRNA reduce SOCE. Furthermore, various molecules that Orai1 regulates have been reported, including Ca^2+^ signals, extracellular signal-regulated kinase 1/2 (ERK), and protein kinase B (Akt), however, these molecules differ depending on the cancer type or the previous articles. Therefore, further mechanistic analysis is considered necessary in the future. We summarized the role of Orai1 in cancers (Table 1). Orai1 is undoubtedly an important molecule that plays a variety of roles depending on the cancer type. Therefore, it will be extremely important to understand the role of Orai1 for developing new cancer treatments. In this article, we described each element of Orai1 and its related items in cancers.

**Table 1:** Reproted roles of Orai1-mediated signaling pathway in cancer

Cancer type	Reported functions	Orai1 expression (compared to normal cells or tissues)	Proposed effector pathway(s)	Pathophysiologic process	Inhibitors/knockdown effect	References [No.]
Oral cancer	Tumorigenicity, tumor sphere formation, proliferation, migration, stemness	Protein↑	NFAT; Lamin B	SOCE	Dominant negative Orai1 mutant (E106Q); Ectopic Orai1 expression	Lee et al. [9]
	Migration, proliferation, colony formation	mRNA↑; protein↑	Akt; mTOR; NF-κB; CXCR4; MMP-9	SOCE	2-APB; LaCl_2_; SKF96365; siRNA	Singh et al. [13]
	Migration/metastasis	N.D	PI3K; ERK; calpain; α-spectrin; MMP-2; MMP-9	Ca^2+^ influx	YM58483; shRNA	Osawa et al. [12]
	Proliferation, migration, invasion, clinical histopathological features	mRNA↑; protein↑	N.D	N.D	siRNA	Wang et al. [14]
Melanoma	Migration/metastasis, proliferation (in part)	Protein↑	ERK; CaMKII; Raf-I; MEK; α-spectrin; calpain	SOCE	YM58483; shRNA	Umemura et al. [7]
	Invadopodium formation, invasion, metastasis	Protein↑	Src	Ca^2+^ oscillations	SKF96365; 2-APB; A23187; shRNA	Sun et al. [36]
	Proliferation, Migration, invasion	Protein↑	MITF; JARID1B; Brn2	SOCE	2-APB; siRNA	Stanisz et al. [8]
	Invasion, metastasis, metabolism	N.D	Glycolysis; cholesterol	UV-induced SOCE	YM58483; UV2; UV5	Gross e al. [22]
Breast cancer	Development, proliferation	mRNA↑	N.D	SOCE	2-APB; siRNA	McAndrew et al. [19]
	Migration, invasion, attachment, angiogenesis	N.D	Notch1/ NFAT4	Hypoxia	SKF96365; DAPT; shRNA	Liu et al. [38]
	EMT	N.D	Vimentin	SOCE	YM58483; EGTA; Cyto D; siRNA	Stewart et al.[25]
Glioblastoma	Invasion, proliferation	mRNA↑(in part)	N.D	SOCE; CRAC currents	siRNA	Motiani et al. [15]
Cervical cancer	Proliferation, colony formation, tumor growth	mRNA↑; protein↑	IL-6	SOCE	SKF96365; AnCoA4	Pan et al. [16]
Multipe myeloma	Proliferation, cell cycle, apoptosis, clinical outcome	mRNA↑; protein↑	N.D	SOCE	SKF96365; DES; 2-APB; siRNA	Wang et al. [17]
Colorectal cancer	EMT, migration, clinical outcome	Protein↑	E-cadherin; N-cadherin; Vimentin; calpain; TGF-β1	SOCE	shRNA; 2-APB	Kang et al. [18]
Pancreatic stellate cell	Proliferation, cell cycle	N.D	Akt; TGF-β1	SOCE	LY294002; siRNA	Radoslavova et al. [20]

*Akt* Protein kinase B, *CaM* calmodulin, *Cyto D* cytochalasin D, *ERK* extracellular signal-regulated kinase, *EMT* epithelial–mesenchymal transition, *IL-6* interleukin-6, *MMP* matrix metalloproteinases, *MTIF* microphthalmia-associated transcription factor, *mTOR* the mammalian target of rapamycin, *NFAT* nuclear factor of activated T-cells, *NF-κB* nuclear factor-kappa B, *PI3K* phosphatidylinositol-3 kinase, *UV* ultraviolet, *N.D.* not determined

## Orai1 is highly expressed in various cancer cells and cardiac fibroblasts

There are many reports about the messenger ribonucleic acid (mRNA) transcription and protein expression of Orai1 in various cancer cells. Indeed, we found that Orai1 was abundantly expressed in human primary melanoma cell lines (WM115 and WM1552 cells), human metastatic melanoma cell lines (SK-Mel-2, C8161, SK- and Mel-24 cells), a human melanocyte cell line (HEMA-LP cells) and a mouse melanoma cell line (B16 cells) [7]. We also examined the expression of Orai1 in human melanoma tissues by a microarray. Orai1 was detected by immunohistochemical (IHC) staining. Similarly, Stanisz et al. reported that Orai1 was expressed in human melanoma cell lines (SK-Mel-28, SK-Mel-5 and WM3734 cells) [8]. They reported that Orai1 was expressed in dermal tumor nests and blood vessel infiltrates of melanoma patients, suggesting that Orai1 is a maker of highly invasive tumor cell types.

Lee et al. demonstrated Orai1 protein expression in normal human oral keratinocytes (NHOK cells), precancerous, nontumorigenic immortalized oral epithelial cell lines (NOK-SI, OKF6/tert, and HOK-16B cells) and human oral squamous cell carcinoma (OSCC) cell lines (HOK-16B-BapT, SCC4, SCC9/TNF, UM17b, and YD38 cells) by western blotting analysis [9]. Orai1 is predominantly localized to the PM, with diffused staining in both the cytoplasm and nucleus, according to IHC staining of patient tissue specimens of human OSCC compared to those of normal human oral keratinocytes (NHOK cells) or oral dysplasia samples. These results are consistent with findings in human melanoma samples.

In 1991, Thun et al. first reported that regular use of aspirin, a cyclooxygenase (COX) inhibitor, at low doses may reduce the risk of patients with fatal colon cancer [10]. Death rates from colon cancer decreased with more frequent aspirin use in both men and women. The effect of COX on arachidonic acid generates prostaglandin E_2_ (PGE_2_), which is a major product and an important factor that causes fever, pain, and inflammation. In our review article, we described that among prostanoids, PGE_2_ is the most widely produced in the body and most broadly distributed [11]. The importance of PGE_2_ and EP4, one of four receptor subtypes for PGE_2_, in various tissues and cancers has been reported.

We reported that both the Orai1 and EP4 proteins were expressed in human OSCC cells (HSC-3 and OSC-19 cells) [12]. Surprisingly, we found that Orai1 colocalized and formed complexes with EP4. Furthermore, Singh et al. demonstrated that Orai1 was highly expressed in human OSCC tissues compared to normal tissues according to IHC staining [13]. Similarly, Wang et al. reported that the mRNA transcription and protein expression of Orai1 in patients with OSCC were significantly enhanced as compared with normal samples according to IHC staining [14].

Motiani et al. demonstrated that Orai1 was also expressed in human glioblastoma (GBM) and essential for human GBM invasion [15]. The authors reported that primary GBM cell lines derived from surgical samples from GBM patients exhibited increased Orai1 mRNA transcription compared to nonmalignant human primary astrocytes (HPA). Pan et al. reported that analysis of RNA-seq data of human cervical cancer patients from The Cancer Genome Atlas (TCGA) revealed that the mRNA level of Orai1 was upregulated compared to the normal cervical tissues [16]. Real-time quantitative polymerase chain reaction (RT-qPCR) showed that mRNA transcription of Orai1 was approximately fivefold higher in 62% (13/21) of the cervical cancer tissues compared to the normal tissues. Furthermore, they evaluated 87 patient cervical cancer tissue samples and 34 normal cervical tissues by IHC staining, and the results showed that Orai1 protein expression was significantly higher in the tumor samples. They concluded that Orai1 was strongly associated with cervical cancer progression.

Wang et al. reported that the expression of Orai1 was associated with the clinical outcome of multiple myeloma (MM) [17]. These authors evaluated the protein expression of Orail1 in bone marrow samples from 60 MM patients and 21 negative controls by IHC staining. They found that the Orai1 protein was abundantly expressed in MM patient bone marrow samples. RT–PCR assay showed that the mRNA transcription of Orai1 was observed in MM cell lines (KM3 and U266 cells). In accordance with human tissue samples, the mRNA transcription of Orai1 was higher in primary MM cells (KM3 and U266 cells) than in cells from healthy controls. Furthermore, western blotting analysis also showed that the Orai1 protein was expressed in MM cell lines (KM3 and U266 cells). The protein expression of Orai1 was higher in MM cell lines and MM patient samples than negative controls.

Kang et al. demonstrated that Orai1 expression was higher in colorectal cancer (CRC) patient tissues than in adjacent noncancerous tissues according to IHC staining and western blotting analysis [18]. They reported that the Orai1 protein was highly expressed in CRC tumors. Furthermore, these authors analyzed the association between the protein expression of Orai1 and the clinicopathological parameters of 80 CRC patients by IHC staining. They concluded that high Orai1 protein expression was highly associated with advanced clinical stage, i.e., high incidence of metastasis.

There are more reports about the relationship between Orai1 and breast cancer compared to other cancers. McAndrew et al. compared the mRNA transcription of Orai1, Orai2 and Orai3 in human breast cell lines. The mRNA transcription of Orai1 was increased by 21-fold in many of the human breast cancer cell lines (T-47D, MDA-MB-231, ZR-75-1, MCF-7, BT-483 and SK-BR-3 cells) compared with the nonmalignant mammary epithelial cell lines (184B5 and 184A1 cells), suggesting that Orai1 was the predominant isoform in the MCF-7, ZR-75-1, MDA-MB-231, and T-47D cell lines [19]. Interestingly, the mRNA levels of Orai2 and Orai3 were similar in breast cancer-derived cell lines and nonmalignant mammary epithelial cell lines. Radoslavova et al. reported that Orai1 was expressed in human pancreatic stellate cells (PS-1 cells and RLTCs), and it was associated with cancer according to western blotting analysis [20]. We confirmed mRNA and protein Orai1 expression in human cardiac fibroblasts (HCFs) by RT‒qPCR and western blotting analysis [21]. Taken together, Orai1 is abundantly expressed in various cancer cells, pancreatic stellate cells and cardiac fibroblast cells.

## SOCE is related to cancer progression

SOCE is a major mechanism by which Ca^2+^ is imported from extracellular space to the intracellular space [1]. It is well known that Ca^2+^ is an essential and ubiquitous second messenger that regulates many different cellular processes [3]. We examined whether SOCE occurs in some human melanoma and melanocyte cell lines. Interestingly, metastatic (SK-Mel-2, C8161, SK-Mel-24 and UACC2577 cells), but not primary (WM3248 and WM1552C cells) melanoma cell lines showed higher SOCE peak amplitudes than a melanocyte cell line (HEMA-LP cells) [7]. Our data suggested that SOCE was enhanced in metastatic melanoma and that the activation of SOCE was related to melanoma progression. Furthermore, Gross et al. reported that exposure to ultraviolet (UV) radiation suppressed SOCE in melanoma (SK-Mel-5, UACC1273, SK-Mel-2, FS13, SK-Mel-28, and UACC257 cells) [22].

Singh et al. reported that OSCC cells (SAS cells) showed significantly more SOCE than normal cells (HaCaT cells) [13]. To study the effect of a tobacco-associated carcinogen on SOCE, Singh et al. used *N*-nitrosonornicotine (NNN) and a synthetic carcinogen 4-nitroquinoline 1-oxide (4-NQO) [13]. It is well known that tobacco is a primary carcinogenic factor. As expected, both agents increased SOCE in SAS cells. Motiani et al. reported that the peak amplitudes of SOCE in human primary GBM cells (GBM1 and GBM8 cells) and human GBM cells (U251-MG cells) was significantly higher than that in human primary astrocytes (HPAs) [15]. This result is consistent with our result.

Next, we introduced evidence related to SOCE inhibitors. We investigated whether a pyrazole compound, YM58483 (BTP2), which is known to inhibit SOCE in nonmelanoma cells, affects SOCE in human melanoma [7, 23, 24]. Indeed, YM58483 suppressed SOCE in metastatic melanoma cell lines (SK-Mel-2 and SK-Mel-24 cells). Furthermore, YM-58483 suppressed the proliferation of melanoma cells (SK-Mel-2, C8161 and SK-Mel-24 cells) in a dose-dependent manner. YM-58483 also significantly decreased the migration of SK-Mel-2 cells. Gross et al. also demonstrated that exposure to ultraviolet (UV) radiation suppressed SOCE and enhanced invasion [22]. Furthermore, YM-58483 suppressed SOCE and promoted invasion in mouse melanoma cells (B16N cells) according to transwell invasion assay. These results were opposite to those of previous reports.

Stewart et al. also reported that YM58483 inhibited SOCE in breast cancer cells (MD-MB-486 cells) in a dose-dependent manner [25]. Sign et al. showed that 2-APB (0.001 to 100 μM), LaCl_3_ (0.1 to 1000 μM) and SKF96365 (1 to 20 μM) decreased the number of OSCC cells (SAS cells) [13]. Wang et al. reported that other SOCE inhibitors (SKF-96365, DES and 2-APB) suppressed the viability of human MM cell lines (KM3 and U266 cells) and primary MM cells in a dose-dependent manner [17]. These inhibitors also significantly increased the proportion of cells in the G2/M phases of the cell cycle and enhanced cell apoptosis after 48 h of treatment. Montiani et al. reported that 5 μM Gd^3+^ and 30 μM 2-APB inhibited SOCE in HPAs and primary GBM cells (GBM1 and GBM8 cells) [15]. Taken together, SOCE has been observed in various cancers. In addition, the degree of SOCE might be a biomarker indicating progression in various cancers.

## Orai1 is highly involved in SOCE in various cancers

Orai1, which is a highly Ca^2+^-selective channel that is in the PM, is a key calcium channel that participates in SOCE. Therefore, we investigated whether Orai1 also plays an important role in SOCE in human melanoma cells. We first established models of human melanoma cells (SK-Mel-2, C8161 ad SK-Mel-24 cells) transduced with Orai1-targeting short hairpin RNA (shRNA) using lentivirus, and then we measured SOCE by Fluo-4AM fluorescence imaging of intracellular Ca^2+^ levels. Silencing of Orai1 attenuated SOCE in human melanoma cells [7]. Stanisz et al. also showed that the silencing of Orai1 by siRNA reduced SOCE in human melanoma cell lines (SK-Mel-5 and WM3734 cells) using Fura-2-based fluorescent Ca^2+^ imaging [8]. Lee et al. demonstrated that Orai1 is required for SOCE in human OSCC cells [9]. They established a dominant negative Orai1 mutant (E106Q) and expressed this mutant in OSCC cells (SCC4 cells) via retroviral vector transduction. Then, silencing of Orai1 decreased SOCE in SCC4 cells.

The silencing of Orai1 by siRNA partially decreased SOCE in human breast cancer cells (MCF-7 and MDA-MB-231 cells) [19]. Complete inhibition was not achieved, so the authors assumed that Orai2 and Orai3 might explain, in part, why SOCE was not completely affected by Orai1 inhibition. Similarly, silencing of Orai1 by siRNA decreased SOCE in MDA-MB-231 cells [26]. Silencing of Orai1 by siRNA decreased SOCE by 66% in human primary GBM cells (GBM_1_ cells) [15]. Silencing of Orai1 by siRNA attenuated SOCE in human cervical cancer cells (Caski or SiHa cells) [16]. Silencing of Orai1 by siRNA partially, but not completely, decreased SOCE in human MM cells (KM3 cells) [17]. Radoslavova et al. demonstrated that silencing of Orai1 by siRNA reduced SOCE compared to control in human pancreatic stellate cells (PS-1 and RLT cells) [20]. Silencing of Orai1 by shRNA decreased Ca^2+^ entry in human CRC cancer cells (SW620 cells) compared with control cells [18]. Taken together, Orai1 is one of the most important molecules for regulating SOCE in various cancers.

## Orai1 regulates multiple signaling pathways in cancer cells via Ca^2+^ influx

Orai1 regulates multiple signaling pathways in cancer cells by regulating the influx of Ca^2+^ from the extracellular space. We found that SOCE promptly phosphorylated the extracellular signal-regulated kinase 1/2 (ERK) signaling molecule in human metastatic melanoma cell lines (C8161 and SK-Mel-24 cells) [7]. The ERK signaling pathway is known to play a major role in malignant transformation and drug resistance of melanoma cells [27, 28]. Indeed, thapsigargin, which is an inhibitor of sarcoplasmic reticulum Ca^2+^-ATPase (SERCA), promptly phosphorylated ERK and promoted α-spectrin cleavage according to western blotting analysis. YM58483 (BTP2), a pyrazole compound, attenuated the thapsigargin-induced phosphorylation of ERK1/2.

The EP4 prostanoid receptors are one of four receptor subtypes for prostaglandin E_2_ (PGE_2_) [11]. As mentioned above, we found that Orai1 colocalized and formed complex with EP4 according to IP [12]. Therefore, we investigated whether an EP4 agonist (ONO-AE1-437) increased the intracellular Ca^2+^ concentrations through Orai1 in human OSCC cells (HSC-3 cells). Indeed, the EP4 agonist promptly increased Ca^2+^ concentrations. In contrast, silencing of either EP4 or Orai1 prevented the EP4 agonist-induced Ca^2+^ increase in HSC-3 cells. Interestingly, silencing of STIM1 did not reverse the effects of the EP4 agonist. These results indicated that EP4 directly activated Orai1 without STIM1. Furthermore, chelating of extracellular Ca^2+^ and YM58483 reversed the EP4 agonist-induced Ca^2+^ increase, but the IP_3_ receptor inhibitor xestospongin C did not have this effect. The phosphatidylinositol-3 kinase (PIK3) inhibitor LY294002 also prevented the EP4 agonist-induced Ca^2+^ increase, but the protein kinase A (PKA) inhibitor PKI-(14–22)-amide did not. Taken together, we concluded that EP4 signaling mediates Ca^2+^ influx from the extracellular space through the PI3K pathway, not the PKA pathway.

Next, we investigated whether the EP4 agonist phosphorylated ERK in HSC-3 cells because we previously confirmed that the thapsigargin-mediated increase in SOCE increased the phosphorylation of ERK in human melanoma cells (C8161 cells) [7]. Indeed, the EP4 agonist promptly increased the phosphorylation of ERK, calpain and cleaved α-spectrin in HSC-3 cells [12]. In contrast, the PIK3 inhibitor LY294002 reversed the EP4 agonist-induced phosphorylation of ERK, but the PKA inhibitor PKI-(14–22)-amide did not have this effect. Furthermore, the silencing of Orai1 by shRNA reversed the EP4 agonist-induced phosphorylation of ERK in HSC-3 cells. Taken together, we concluded that EP4 activated Orai1 via PI3K and then induced Ca^2+^ influx from the extracellular space through Orai1, resulting in the phosphorylation of ERK. Therefore, we were surprised that Orai1 was activated by EP4, which is coupled to Gαs and Gαi, through PI3K (rather than phospholipase C), resulting in the induction of a Ca^2+^ influx from the extracellular space in a STIM1-independent manner.

Singh et al. reported that silencing of Orai1 by siRNA decreased the phosphorylation of the serine-threonine kinase Akt (known also as protein kinase B) at both the Thr 308 and Ser 473 residues as well as the phosphorylation of nuclear factor-kappa B (NF-κB) in human OSCC cells (SAS cells) according to western blotting analysis [13]. Furthermore, silencing of Orai1 decreased the expression of the chemokine receptors, chemokine receptors CXCR4 and matrix metalloproteinase (MMP)-9. Bong et al. reported that silencing of PTEN (the negative PI3K regulator/tumor suppressor) or treatment with the pharmacological Akt activator SC79 resulted in the phosphorylation and activation of Akt, which is the downstream mediator of PI3K, but total Akt levels were unaffected [26]. The activation of Akt increased Ca^2+^ entry via Orai1 in human breast cancer cells.

## Orai1 coordinates other molecules in cancer

As mentioned above, there are many reports that Orai1 regulates Ca^2+^ entry from the extracellular space and cell invasion in cancers. Our previous review demonstrated that PGE_2_ and EP4, which is one of four receptor subtypes for PGE_2_, regulate cell proliferation, cell cycle arrest, polyp formation, migration, invasion, apoptosis and adhesion in various cancers [11]. Therefore, we hypothesized that EP4 regulated physiological functions by increasing Ca^2+^ concentrations. Therefore, we investigated whether an EP4 agonist increased the intracellular Ca^2+^ concentrations. As expected, an EP4 agonist (ONO-AE1-437) promptly increased the Ca^2+^ concentrations in OSCC cells (HSC-3 cells), GBM cells (LN229 cells) and human breast cancer cells (MCF7 cells). The chelation of extracellular Ca^2+^ and treatment with YM58483 reversed the EP4 agonist-induced increases in Ca^2+^ concentration. Therefore, we assumed that the source of Ca^2+^ is the extracellular space.

Next, we investigated whether Orai1 contributed to the EP4-induced Ca^2+^ influx. We established Orai1-knockdown HSC-3 cells by shRNA transduction. The silencing of Orai1 reversed the EP4 agonist-induced Ca^2+^ influx. We concluded that Orai1 participated in the EP4-induced increases in intracellular Ca^2+^ levels in human OSCC cells [12]. In this article, we reported that Orai1 formed a complex with EP4 and transient receptor potential channel (TRPC)1, but not with STIM1, in human OSCC cells (HSC-3). As far as we know, this is the first report that EP4 regulates Ca^2+^ concentrations through Orai1. However, whether EP4 also regulates Ca^2+^ through Orai1 in other cancers remains elusive. To further confirm the mechanism of EP4-induced Ca^2+^ elevation in other cancers, we used other cancer cell lines except OSCC cells and perfume the same experiment.

Jardin et al. observed a dynamic interaction between endogenously expressed human TRPC6 with either Orai1 and STIM1 or with human TRPC3, allowing it to participate in the activation of capacitive Ca^2+^ entry (CCE) pathways [29]. It is known that TRPC3 is also an SOCE component [30]. In contrast, Liao et al. demonstrated by IP that Orai1 physically interacts with the N and C termini of TRPC3 and TRPC6 [31]. Therefore, we focused on the function of TRPC3 with respect to its association with Orai1. We confirmed the TRPC3 was abundantly expressed in human melanoma tissues according to a tissue microarray [32]. The mRNA transcription of TRPC3 was observed in SK-Mel-2 cells (expressing mutant NRAS), SK-Mel-187 (metastatic human melanoma cell line expressing wild-type BRAF), SK-Mel-24 (expressing mutant BRAFV600E), C8161 (metastatic human melanoma cell line expression wild-type BRAF), and HEMA-LP (melanocyte cell line). Indeed, we confirmed that a pyrazole compound Pyr3, which is a TRPC3-selective inhibitor, suppressed SOCE in human melanoma cells (C81619 cells) [32, 33]. The silencing of TRPC3 decreased the proliferation of C8161 cells over 48 h. Additionally, in C8161 cells, the silencing of TRPC3 decreased migration according to time-lapse video recording and MMP-9 secretion according to gelatin zymography. It is well known that MMP-9, a gelatinase, dissolves type IV collagen in basal membranes and enhances the cellular migration of melanoma [34]. Recently, Sánchez-Collado et al. reported that the interaction between Orai1 channels and the Ca^2+^-sensitive adenylyl cyclase 8 (AC8) plays an important role both in the activation of the cyclic adenosine monophosphate (cAMP)/PKA signaling pathway and the modulation of Orai1-dependent Ca^2+^ signaling [35]. Taken together, Orai1 coordinates various molecules in cancers.

## Orai1 is involved in cancer progression in vitro

Intracellular Ca^2+^ is essential for the migration of various types of cells, including cancer cells. We previously demonstrated that the transduction of Orai1-targeting shRNA into human melanoma cells (SK-Mel-2 and SK-Mel-24 cells) using lentivirus decreased cell migration according to boyden chamber assays as well as migration distance according to time-lapse video recording [7]. Interestingly, silencing of Orai1 inhibited the proliferation of C8161 cells but not that of SK-Mel-2 cells or SK-Mel-24 cells. Silencing of Orai1 also decreased the number of lamellipodia, which reflects actin assembly/disassembly activity. Taken together, Orai1 plays important roles in melanoma cell proliferation and migration. Sun et al. demonstrated that silencing of Orai1 by shRNA decreased the mean number of invadopodia per cell and the area of gelatin degradation per cell in human primary melanoma cells (WM793 cells) [36]. Furthermore, silencing of Orai1 by shRNA significantly decreased the frequency of Ca^2+^ oscillation compared to the control. Additionally, Stanisz et al. reported that silencing of Orai1 decreased the collagen invasion of a human primary melanoma cell line (WM3734 cells) and human metastatic melanoma cell line (SK-Mel-5 cells) [8]. Additionally, the CRAC channel inhibitor 2-APB exerted similar effects.

Lee et al. reported that silencing of Orai1 with a retroviral vector expressing E106Q (dominant negative Orai1) slightly decreased the number of human OSCC cells (SCC4 cells) and significantly decreased the formation of colonies in soft agar [9]. The dominant negative Orai1 mutant (E106Q) inhibited the tumor sphere forming of human SCC cells (SCC4 cells) and human oral keratinocytes (HOK-16B BapT cells) according to tumor sphere formation assay. Additionally, E106Q expression decreased the activity of aldehyde dehydrogenase1 (ALDH1), which is one cancer stem-like cell (CSC) marker that is known to enrich CSCs in solid malignancies, including head and neck cancer [37]. Because a key feature of CSCs is their self-renewal capacity, the authors demonstrated that Orai1 was essential for sustaining the self-renewal capacity of CSCs. In contrast, the authors overexpressed Orai1 in nontumorigenic immortalized oral epithelial cells (HOK-16B cells) via a lentiviral vector expressing Orai1. Orai1 overexpression led to significant increases in the proliferation of HOK-16B cells, suggesting that Orai1 endowed nontumorigenic oral epithelial cells with tumorigenic potential.

Singh et al. demonstrated that silencing of Orai1 inhibited the migration and reduced the colonization of OSCC cells (SAS cells) according to a wound-healing assay and a colony formation assay [13]. Motiani et al. reported that silencing of Orai1 by siRNA decreased the proliferation of human GBM cells (GBM1 and GBM8 cells) according to MTT assay, but no effect was observed in HPAs [15]. These authors also reported that silencing of Orai1 reduced the invasion of GBM1 and GBM8 cells in matrigel invasion assays, but similar effects were not observed in HPAs. Pan et al. demonstrated that silencing Orai1 significantly decreased the secretion of interleukin (IL)-6 by human cervical cancer cells (Caski and SiHa cells) [16]. Furthermore, they also demonstrated a positive correlation between Orai1 and IL-6 expression in human cervical cancer samples.

There are more reports about the relationship between Orai1 and breast cancer than other cancers. Orai1 is critical for notch-driven aggressiveness of triple-negative breast cancers under hypoxic conditions [38]. In contrast, Sanchez-Collado et al. reported that Orai2 regulated SOCE and cell cycle progression in human breast cancer cells [39]. Wang et al. demonstrated that silencing of Orai1 by siRNA triggered the typical nuclear features of apoptosis, including the pyknosis of cell nuclei and the appearance of apoptotic bodies with nuclear membranes, in human MM cells (KM3 cells) as shown by transmission electron microscopy (TEM) [17]. Furthermore, in KM3 cells, silencing of Orai1 by siRNA also inhibited cell viability according to trypan blue exclusion assay, promoted apoptosis and significantly increased the proportion of cells in the G2/M phases of the cell cycle compared to the control siRNA group.

Radoslavova et al. demonstrated that silencing of Orai1 by siRNA decreased the cell proliferation and induced cell cycle arrest in the G0/G1 phase, followed by a decrease in the number of cells in the S phase without affecting the number of cell sin the G2/M phase in human pancreatic stellate cells (PS-1 and RLT cells), which are crucial mediators of pancreatic desmoplasia [20]. Kang et al. reported that human CRC cells (SW620 cells) that were transfected with Orai1-targeting shRNA showed highly organized cell‒cell adhesion and cobblestone shapes, and an epithelioid morphology appeared [18]. Additionally, they showed that the expression of E-cadherin was significantly upregulated the expression of N-cadherin and vimentin was obviously downregulated in SW620-shOrai1 cells compared with the control cells according to western blotting analysis. These results suggested that Orai1 might regulate the expression of EMT-relevant molecules.

## Orai1 is involved in cancer progression in vivo

There are many reports that Orai1 regulates tumor growth, cell migration, metastasis and EMT. We established human melanoma cells transduced with Orai1-targeting shRNA using lentivirus. Silencing of Orai1 decreased the numbers of metastatic colonies in the lungs of mice, suggesting that Orai1 regulates the metastasis of melanoma in vivo [7]. As mentioned above, we found that Orai1 formed a complex with EP4 in OSCC cells according to IP [12]. Indeed, the EP4 agonist significantly increased the intracellular Ca^2+^ concentrations in human OSCC cells (HSC-3 cells), human GBM cells (LN229 cells), and human breast cancer cells (MCF7 cells). Silencing of EP4 or Orai1 reversed the EP4-mediated increase in Ca^2+^ concentration in two types of knockdown cells (HSC-3 cells). PGE_2_ and EP4 agonist promoted the migration of HSC-3 cells. In contrast, an EP4 antagonist inhibited the PGE_2_-induced migration of HSC-3 cells. Therefore, we hypothesized that EP4 increased the intracellular Ca^2+^ concentration via Orai1 and then promoted the migration of OSCC cells. We first established EP4-knockdown OSCC cells (HSC-3 cells) by shRNA and then injected them into the tail veins of BALB/c nu/nu mice. Silencing of EP4 suppressed the metastasis of oral cancer into the lungs of mice, suggesting that EP4 regulates intracellular Ca^2+^ levels via Orai1 and promotes cell migration, resulting in lung metastasis.

Lee et al. reported that Orai1 is required for the tumorigenicity of OSCC cells [9]. To investigate the role of Orai1 in OSCC growth, these authors established Orai1-knockdown OSCC cells (SCC4 cells) by expressing a dominant negative Orai1 mutant (E106Q) via retroviral vector. The Orai1 mutant (E106Q) inhibited xenograft tumor formation in the nude mice. In contrast, the authors overexpressed Orai1 in nontumorigenic immortalized oral epithelial cells (HOK-16B cells) with a lentiviral vector expressing Orai1. They examined the effect of Orai1 on tumor growth in vivo. Three out of 5 mice injected with HOK16B/Orai1 developed tumor growth, but similar results were not observed in the control group that was injected with cells carrying an empty vector.

Pan et al. reported that silencing of Orai1 in human cervical cancer cells (Caski and SiHa cells) by siRNA resulted in the formation of significantly smaller tumors (both in terms of volume and weight) compared to those that formed from control cells in vivo [16].

## Orai1 knockdown attenuated doxorubicin-induced cardiac toxicity

Doxorubicin (DOX) causes cardiotoxicity and induces cardiac dysfunction [40]. Many studies have identified mechanisms underlying DOX-induced cardiotoxicity, including oxidative stress, deoxyribonucleic acid (DNA) intercalation, topoisomerase II inhibition, apoptosis, mitochondrial dysfunction, autophagy, ferroptosis, inflammatory cytokines, and calcium homeostasis [41]. DOX also induces the trans-differentiation of cardiac fibroblasts into myofibroblasts and increases the expression levels of MMP1, IL-6, transforming growth factor-β (TGF-β), and collagen in HCFs [42].

Orai1 and SOCE are highly active in HCFs [43]. Orai1 expression is upregulated in cardiac ventricular fibroblasts from patients with heart failure (HF) compared with those of patients without HF. Similar trends are not observed for STIM1 expression. As mentioned above, many reports have demonstrated that Orai1 is a key player in cell apoptosis. Therefore, we hypothesized that Orai1 plays an important role in the pathogenesis of DOX-induced HF. In particularly, we focused on HCFs because HCFs account for 60–70% of the cells in the heart. We confirmed the relationship between Orai1/SOCE and DOX-induced HF in HCFs [21]. Both YM-58483 (BTP2), which is an Orai1/SOCE inhibitor, and siRNA-mediated Orai1 knockdown attenuated DOX-induced p53 protein expression and DOX-induced apoptosis, according to terminal transferase dUTP nick end labeling (TUNEL) staining, in HCFs cells. Furthermore, YM-58483 significantly attenuated DOX-induced apoptosis in the hearts of mice.

Lithium has been recommended as the first-line treatment for mood symptoms in patients with bipolar disorder [44]. Lithium (LiCl) (0.1 mM) decreased the protein expression of Orai1 in HCFs. Additionally, therapeutic trough levels of lithium suppressed the Ca^2+^ release from the ER and SOCE, resulting in a reduction in cell migration and collagen synthesis in HCFs [45].

Taken together, Orai1 is associated with not only cancer cells, but also non-cancer cells, e.g., cardiac fibroblast. Further studies regarding the similarities and differences of Orai1 function in cancer cells and non-cancer cells.

## Orai1-deficient mice

There are few reports about the relationship between Orai1-deficiency in mice and cancer. Therefore, we summarized the evidence characterizing Orai1-deficient mice. Gwack et al. generated mice with targeted disruption of the Orai1 gene [46]. IHC staining analysis showed that Orai1 is expressed in lymphocytes, skin, hair follicles and muscle of wild-type mice, but it is not expressed in Orai1^(−/−)^ mice. Importantly, knocking out Orai1 in mice on the inbred C57BL/6 background resulted in perinatal lethality. The researchers overcame this limitation by crossing these mice to outbred ICR (Crl:CD1) mice. The authors reported that Orai1^(−/−)^ mice were small in size, with eyelid irritation and sporadic hair loss resembling the cyclical alopecia observed in mice with keratinocyte-specific deletion of the Cnb1 gene, which encodes Calcineurin subunit Yang et al. also reported that mice with ubiquitous Orai1 deficiency show early lethality [47]. They generated a platelet-specific Orai1-deficient mouse line by mating Orai1^flox/flox^ mice with Pf4-Cre mice [48].

Vig et al. generated Orai1-deficient mice (Cracm1^−/−^) with gene-trap technology [49]. Interestingly, Orai1-deficient mice are much smaller than wild type mice. This report is consistent of Gwack’s report. Whole-body necropsy and extensive histological analysis of Orai1-deficient mice showed no obvious abnormalities. Mast cells derived from Orai1-deficient mice exhibited grossly defective degranulation and cytokine secretion. Furthermore, the allergic reactions in these mice were inhibited.

## Conclusion

Orai1 plays important roles in regulating physiological and pathological functions via Ca^2+^ in cancers. Therefore, Orai1 may be a novel potential target for the treatment of cancers.

## Data Availability

This article is distributed under the terms of the Creative Commons Attribution 4.0 International License (http://creativecommons.org/licenses/by/4.0/), which permits unrestricted use, distribution, and reproduction in any medium, provided you give appropriate credit to the original author(s) and the source, provide a link to the Creative Commons license, and indicate if changes were made.

## Abbreviations

AC: Adenylyl cyclase
Akt: Protein kinase B
cAMP: Cyclic adenosine monophosphate
COX: Cyclooxygenase
CRAC: Ca^2+^ release-activated Ca^2+^ channels
CRC: Colorectal cancer
CSC: Cancer stem-like cell
DOX: Doxorubicin
EMT: Epithelial–mesenchymal transition
ER: Endoplasmic reticulum
ERK: Extracellular signal-regulated kinase 1/2
GBM: Glioblastoma multiforme
GPCR: G protein-coupled receptor
HCFs: Human cardiac fibroblasts
HF: Heart failure
IHC: Immunohistochemical
IL: Interleukin
IP: Immunoprecipitation
IP_3_: Inositol 1, 4, 5-trisphosphate
MM: Multiple myeloma
MMP: Matrix metalloproteinase
mRNA: Messenger ribonucleic acid
Orai1: Orai calcium release-activated calcium modulator 1
OSCC: Oral squamous cell carcinoma
PAH: Pulmonary arterial hypertension
PGE_2_: Prostaglandin E_2_
PHA: Primary human astrocytes
PI3K: Phosphatidylinositol-3 kinase
PKA: Protein kinase A
PM: Plasma membrane
RNA: Ribonucleic acid
RT-qPCR: Real-time quantitative polymerase chain reaction
SCID: Severe combined immune deficiency
SERCA: Sarcoplasmic reticulum Ca^2+^-ATPase
shRNA: Short heparin RNA
siRNA: Small interfering RNA
SOCE: Store-operated-calcium entry
STIM1: Stromal interaction molecule 1
TCGA: The Cancer Genome Atlas
UV: Ultraviolet
